# Clinical Features and Mortality Associated with Severe Malaria in Adults in Southern Mauritania

**DOI:** 10.3390/tropicalmed6010001

**Published:** 2020-12-22

**Authors:** Boushab Mohamed Boushab, Mohamed Salem Ould Ahmedou Salem, Ali Ould Mohamed Salem Boukhary, Philippe Parola, Leonardo Basco

**Affiliations:** 1 Department of Internal Medicine and Infectious Diseases, Kiffa Regional Hospital, Assaba, Mauritania; bboushab@gmail.com; 2 Unité de Recherche Génomes et Milieux, Faculté des Sciences et Techniques, Université de Nouakchott Al-Aasriya, Nouveau Campus Universitaire, BP 5026, Nouakchott, Mauritania; salem0606@yahoo.fr (M.S.O.A.S.); alimedsalem@gmail.com (A.O.M.S.B.); 3 Institut de Recherche pour le Développement (IRD), Aix-Marseille Université, IRD, AP-HM, SSA, VITROME, 13005 Marseille, France; philippe.parola@univ-amu.fr; 4 Institut Hospitalo-Universitaire (IHU)-Méditerranée Infection, 13005 Marseille, France

**Keywords:** artesunate, drug resistance, malaria, quinine, rapid diagnostic test, *Plasmodium falciparum*, Sahel

## Abstract

Severe malaria in adults is not well-studied in Sahelian Africa. Clinical features and mortality associated with severe *Plasmodium falciparum* malaria in adult patients hospitalized in Kiffa, southern Mauritania, were analysed. Patients over 15 years old admitted for severe malaria between August 2016 and December 2019 were included in the present retrospective study. The World Health Organization (WHO) criteria were used to define severe malaria. The presenting clinical characteristics and outcome were compared. Of 4266 patients hospitalized during the study period, 573 (13.4%) had a positive rapid diagnostic test for malaria, and 99 (17.3%; mean age, 37.5 years; range 15–79 years; sex-ratio M/F, 2.1) satisfied the criteria for severe malaria. On admission, the following signs and symptoms were observed in more than one-fourth of the patients: fever (98%), impairment of consciousness (81.8%), multiple convulsions (70.7%), cardiovascular collapse (61.6%), respiratory distress (43.4%), severe anaemia ≤ 80 g/L (36.4%), haemoglobinuria (27.3%), and renal failure (25.3%). Patients were treated with parenteral quinine or artemether. Fourteen (14.1%) patients died. Multiple convulsions, respiratory distress, severe anaemia, haemoglobinuria, acute renal failure, jaundice, and abnormal bleeding occurred more frequently (*p* < 0.05) in deceased patients. Mortality due to severe falciparum malaria is high among adults in southern Mauritania. An adoption of the WHO-recommended first-line treatment for severe malaria, such as parenteral artesunate, is required to lower the mortality rate associated with severe malaria.

## 1. Introduction

Malaria is one of the major public health problems in Mauritania [1]. *Plasmodium falciparum* infections occur mostly in the Sahelian south of the country, where transmission is seasonal (generally from July to October or November) and malaria represents the first cause of outpatient consultation (25%), hospitalization (35.5%), and mortality (39%) [2]. By contrast, *Plasmodium vivax* infections predominate in the Saharan northern region of the country, including Nouakchott, the capital city, and Atar, an oasis city [3,4].

Clinical manifestations of malaria disease vary between children and adults [5]. Signs and symptoms in patients can range from mild febrile syndrome to life-threatening conditions with severe anaemia, pulmonary oedema, metabolic acidosis, acute renal failure, and/or cerebral malaria [6]. Malaria infection may also be asymptomatic in an unknown proportion of human populations in an endemic area. On a global scale, a small proportion of malarial infections lead to severe and complicated malaria, mostly in African children [7]. The underlying mechanisms of pathogenesis are poorly understood. Current therapy of severe malaria relies primarily on rapid parenteral administration of antimalarial drugs and intensive supportive care.

Although severe malaria cases are commonly observed in the Sahelian regions of the country, there are no published clinical studies on severe malaria in Mauritanian adults, except for a single case report from Aioun El Atrouss [8]. The aim of the present study was to describe the epidemiological and clinical aspects of severe and complicated malaria in Mauritanian adults in Kiffa, situated in southern Mauritania, and compare the presenting clinical manifestations and clinical outcome. 

## 2. Patients and Methods 

### 2.1. Study Site

**Figure 1:**
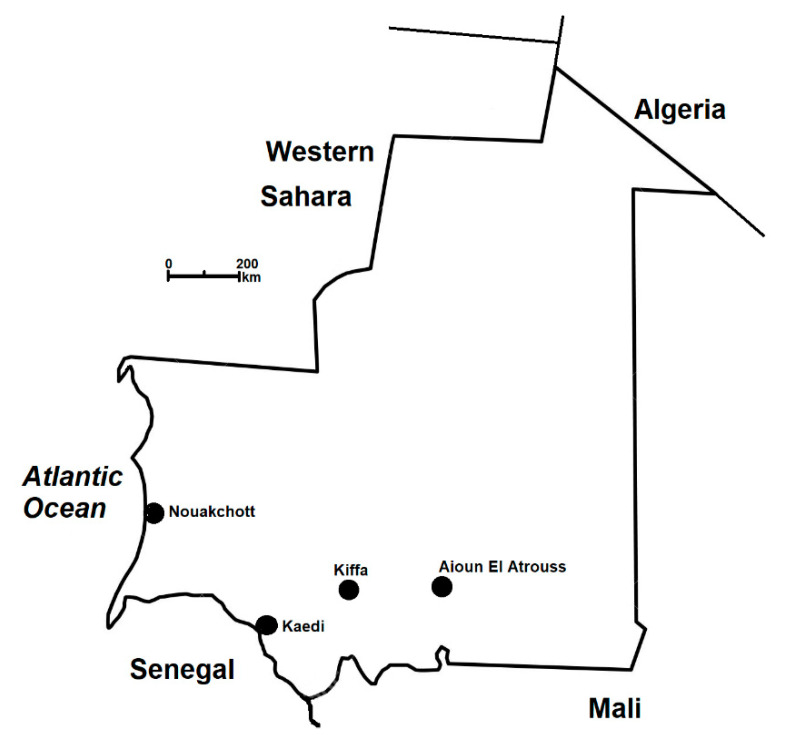
Map showing the location of the study site (Kiffa) in southern Mauritania. Previous studies on severe malaria were conducted in Kaedi and Aïoun El Atrouss [8,11,12]. Nouakchott is the capital city of Mauritania.

Kiffa (16°37’N 11°24’W) is the third largest city of the country, located in the Assaba region in southern Mauritania, approximately 600 km southeast of the capital city, Nouakchott (Figure 1). According to the latest official population census in 2013, there were 325,896 inhabitants in the Assaba region and 50,208 inhabitants (20,632 children aged less than 15 years old; 26,411 adults aged 15–59 years old; and 3165 seniors aged > 60 years old) in the city of Kiffa [9]. The mean minimum and maximum temperatures between January and December were 21.0 °C and 30.5 °C (range, 10.4–46.1 °C) in 2016, 23.1 °C and 36.6 °C (range, 11.5–46.8 °C) in 2017, 17.6 °C and 32.1 °C (range, 9.9–47.1 °C) in 2018, and 24.9 °C and 37.2 °C (range, 18–43 °C) in 2019, respectively [10]. The total annual rainfall was 339 mm, 250 mm, 327 mm, and 347 mm in 2016, 2017, 2018, and 2019, respectively. Rainfall occurred only between June and September or October (peak in July or August). 

### 2.2. Patients

A retrospective study was performed in adult patients with severe and complicated malaria hospitalized between August 2016 and December 2019 based on individual patient records, including socio-demographic information, clinical history, presenting signs and symptoms, laboratory examinations, and outcome. Although a retrospective study design has several disadvantages, in the present study, the fact that clinical information was recorded by the same attending physician tends to reduce selection and information bias. Moreover, we considered the retrospective design to be more appropriate in a situation where reliable data were unavailable from an earlier study in the Mauritanian population. All malaria-infected patients with severe manifestations and without any concomitant chronic disease were hospitalized in the wards of the Department of Internal Medicine and Infectious Diseases of Kiffa Regional Hospital. This is the largest hospital outside the capital city of Nouakchott with a capacity of 150 beds. The hospital conducts an average of 43,000 medical consultations and 2700 surgical operations every year. About 8000 patients are hospitalized every year. Most patients in the eastern region of the country requiring tertiary care are referred to and hospitalized at Kiffa Regional Hospital.

The inclusion criteria were the following: patients of both sexes; at least 15 years old; without an upper age limit; laboratory-confirmed diagnosis of malaria by a rapid diagnostic test (RDT; OptiMAL-IT, Bio-Rad, Marnes-la-Coquette, France); resident in Mauritania for at least two years; and admission to the Department of Internal Medicine and Infectious Diseases of the Kiffa Regional Hospital. Severe malaria was defined according to the World Health Organization (WHO) criteria, namely, the presence of *P. falciparum* in the peripheral blood in association with one or more major criteria of severity [6]. The criteria of “outline bedside clinical classification of severe malaria in adults” and “epidemiological and research definition of severe falciparum malaria” proposed by the WHO were used. Malaria-infected patients with underlying severe chronic cardiac, renal, or hepatic diseases or human immunodeficiency virus/acquired immunodeficiency syndrome, which may interfere with the evolution of malaria, and pregnant women were excluded from analysis. For parasitological diagnosis on admission (day 0) and follow-up, a RDT for malaria was performed. Hyperparasitaemia was not assessed due to limited laboratory resources to perform microscopic examination of blood smear.

Patients were treated with intravenous infusion of quinine (8 mg base/kg every 8 h, equivalent to 10 mg quinine salt/kg every 8 h), as recommended by the Mauritanian malaria control unit [13]. Loading dose of quinine is not recommended in Mauritania. If a patient presented with haemoglobinuria, intramuscular artemether (3.2 mg/kg/day on day 0, followed by 1.6 mg/kg/day until the patient improved) was administered, as recommended by the WHO [6]. When the patients were able to swallow, parenteral treatment was discontinued, and a standard three-day oral treatment with artemisinin-based combination therapy (ACT; artesunate-amodiaquine or artemether-lumefantrine) was prescribed.

### 2.3. Statistical Analysis

Data entry and analysis were performed using Epi-info 3.5.3 software [14]. Comparison of qualitative variables was performed using Fisher’s exact test. Quantitative variables were compared using the Student’s test. The threshold of significance was set at 5%.

### 2.4. Research Ethics

The study was approved by the institutional ethics review committee of the University of Nouakchott Al Aasriya, Nouakchott, Mauritania (approval no. 112/12-09-2014/USTM, 003/2020/CE/UNA) and the Institutional Review Board of the Institut de Recherche pour le Développement (IRD), Marseille, France (Comité consultatif de déontologie et d’éthique approval 15/12/2012).

## 3. Results

**Figure 2:**
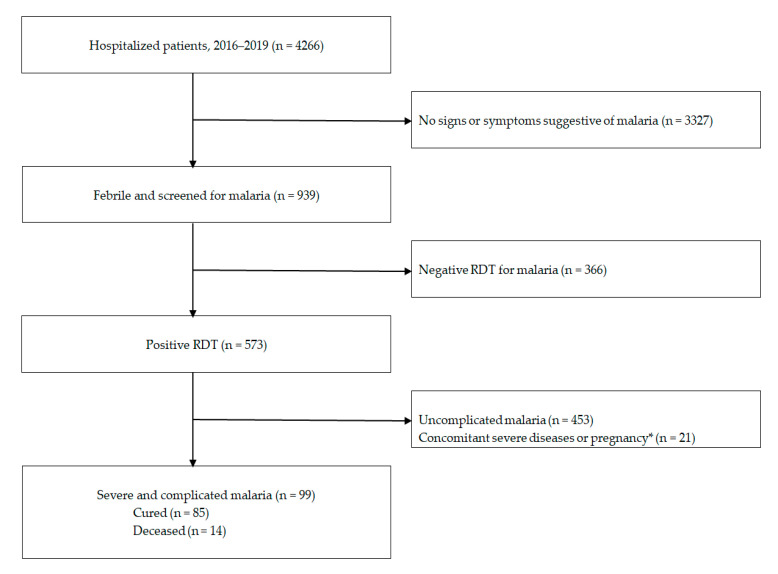
Flow chart of patient recruitment in Kiffa Regional Hospital, southern Mauritania. * These malaria-infected patients also had severe chronic cardiac, renal, or hepatic diseases or human immunodeficiency virus/acquired immunodeficiency syndrome (n = 17) or were pregnant (n = 4). RDT, rapid diagnostic test for malaria.

A total of 4266 patients were hospitalized at Kiffa Regional Hospital, of whom 939 (22.0%) were febrile and screened for malaria during the study period (Figure 2). RDT was positive in 573 of 939 (61.0%) febrile patients admitted to the hospital. Severe malaria was diagnosed in 99 of 573 (17.3%) malaria-infected patients. Fever was present at the time of consultation in 97 (98.0%) patients with severe malaria. Two patients were afebrile on admission (36.5 and 37.3 °C) but reported high-grade fever less than 48 h before hospitalization. The mean (±standard deviation [SD]) body temperature of patients with severe malaria was 39.8 ± 0.7 °C (range, 36.5–41.9 °C). Of patients with severe malaria, 67 (67%) were male and 32 (32%) were female, with a sex ratio (M/F) of 2.1. The mean age (±SD) was 37.5 ± 16.4 years, with a range between 15 and 79 years. Many of the patients (38.4%) were residents of Kiffa city, but the majority (61.6%) came from the suburban area, that is, from surrounding villages.

**Figure 3:**
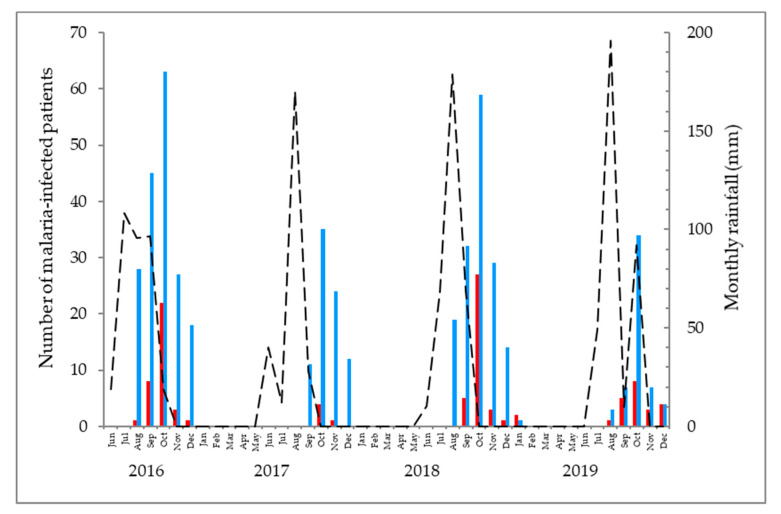
The number of patients with uncomplicated or severe malaria admitted in Kiffa Regional Hospital, southern Mauritania, in 2016–2019 (left vertical axis, coloured bars). The hospital opened on 1 August 2016. Laboratory-confirmed malaria cases were seen from August to December in 2016–2019. Rainfall occurred only between June and September or October in 2016–2019 (right vertical axis, broken line). Blue bar, uncomplicated malaria; red bar, severe malaria; broken line, monthly rainfall (mm).

During the four-year period, similar numbers of severe malaria were observed in 2016 (n = 35) and 2018 (n = 36). Most cases of severe malaria occurred in October (Figure 3). The mean time (±SD) of delay between the onset of symptoms and hospitalization was 3.1 ± 1.0 days (range: 1–9 days). Self-medication with an antimalarial drug (sulfadoxine-pyrimethamine or ACT) was reported by 31 (31.3%) patients. Before referral for hospitalization, 37 (37.4%) patients had medical consultation in town. The mean time delay (±SD) between initial therapeutic intervention and hospitalization in these 37 patients was 3.3 ± 1.7 days, whereas the mean time delay was 2.9 ± 1.4 days in the other patients (*p* > 0.05). An antimalarial treatment was prescribed in 28 of these patients based on a presumptive diagnosis of malaria: 17 received intravenous quinine at an inappropriate dose, and 11 received an oral treatment with other drugs (chloroquine, sulfadoxine-pyrimethamine, ACT).

The clinical manifestations of severe malaria were dominated by fever (98%), impaired consciousness (81.8%), and multiple convulsions (70.7%) (Table 1). The other clinical and laboratory features observed in the patients included cardiovascular collapse (61.6%), respiratory distress (43.4%), severe anaemia ([defined as ≤8.0 g/dL], 36.4%), haemoglobinuria (27.3%), acute renal failure (25.3%), hypoglycaemia (13.1%), jaundice (12.1%), and abnormal bleeding (7.1%). Some of the more commonly observed manifestations (convulsions and cardiovascular collapse) tended to occur more frequently (*p* < 0.05) in men, while other manifestations (impaired consciousness, respiratory distress, and haemoglobinuria) did not show any gender differences. There was no difference in the clinical manifestations with respect to age. The mean haemoglobin (±SD) was 8.6 ± 2.1 g/dL, with a range between 2.3 and 13.0 g/dL.

Seventy-two patients (72.8%) were treated with intravenous quinine (8 mg base [equivalent to 10 mg salt]/kg body weight every 8 h), followed by ACT as soon as they were capable of swallowing. Intramuscular artemether (3.2 mg/kg/day on day 0, followed by 1.6 mg/kg/day until the patient improved) was administered as the first-line treatment in 27 (27.3%) patients presenting with haemoglobinuria on admission.

The mean duration of hospitalization was 3.3 ± 1.3 days (range, 1 to 9 days) (excluding deceased patients). The standard three-day dose of artesunate-amodiaquine or artemether-lumefantrine was prescribed when the patient was capable of swallowing or at hospital discharge. Patients were followed for an average of three weeks after discharge. All surviving patients had a negative RDT at the end of follow-up. One patient developed neurologic sequelae (right hemiplegia). The brain scan showed ischaemic lesion.

Fourteen (14.1%) patients died between 1 and 5 days after hospital admission. Convulsions, respiratory distress, severe anaemia, haemoglobinuria, acute renal failure, jaundice, and abnormal haemorrhage were more frequently observed (*p* < 0.05) in deceased patients, compared to those who were cured (Table 1). More women (n = 9) died than men (n = 5) (*p* = 0.011). Five deceased patients were aged between 15 and 25 years (mean ± SD, 19.2 ± 4.3 years), and nine patients who died were more than 40 years old (mean ± SD, 58.8 ± 17.0; range, 40–79 years old). Age was not associated with higher mortality (*p* = 0.087). Self-medication or inappropriate treatment prior to hospitalization was also not associated with clinical outcome (*p* = 1.0). Thirteen patients treated with quinine died, while only one patient treated with artemether died (*p* = 0.104).

## 4. Discussion

The present study showed a relatively high prevalence of RDT-confirmed malaria among hospitalized patients during the study period (August 2016–December 2019). The prevalence of laboratory-confirmed malaria among febrile hospitalized patients was 61.0% (573/939), and severe malaria was observed in 17.3% (99/573) of malaria-infected patients. Malaria was diagnosed mostly during the transmission season (August–December), with few cases diagnosed in January. The present data on malaria prevalence cannot be compared to previous data in the region because earlier data had been mostly based on presumptive diagnosis of malaria. However, the observations of the present study lend support to the long-held presumption that malaria is the first cause of outpatient consultation and hospitalization in southern Mauritania [3].

Severe malaria accounted for 17.3% of RDT-confirmed malaria seen at the hospital in Kiffa. Very few clinical studies have been conducted on severe malaria in children and adults in Mauritania. In an earlier retrospective study conducted in 2000–2002 in Kaédi (Gorgol region), located along the Senegal River 230 km (508 km by route) southwest of Kiffa, the WHO definition of severe malaria was not applied, but it was reported that severe malaria occurred in 480/722 (66.5%) children and adults with presumptive diagnosis of malaria and that coma and convulsions occurred in 53/722 (7.3%) and 77/722 (10.7%), respectively [11]. Those patients were treated with parenteral quinine. Forty-three (6.0%) patients died. In that study, however, malaria diagnosis was not confirmed in a large majority of patients, and clinical manifestations were not stratified according to age, rendering comparison of results with those of the present study difficult. Another study conducted in 2016 in Aïoun El Atrouss, situated 188 km (218 km by route) to the east of Kiffa, was exclusively in children aged <15 years [12]. A direct comparison of these studies on severe malaria conducted in southern Mauritania (Kiffa, Aïoun El Atrouss, and Kaédi) cannot be made because of the differences in patient population (adults vs children) and diagnostic procedures (microscopy or RDT vs. presumptive diagnosis). Moreover, different definitions of severe malaria have often been used in studies conducted in Africa [15,16,17,18,19,20,21]. Despite these limitations, it can be reasonably argued that severe malaria does occur in Mauritanian adults, mostly in southern Sahelian and Saharo-Sahelian regions where *P. falciparum* transmission has been known to occur for decades. *P. falciparum* malaria also occurs in the city of Nouakchott. However, there are at present no clinical data on severe malaria occurring in adults residing in Nouakchott. Likewise, there is still no evidence that *P. vivax* infection causes severe malaria in the country.

In the present study, 99 cases of severe malaria were admitted for hospitalization over a period of 41 months, corresponding to 2.3% of hospital admissions in Kiffa during the study period. The prevalence of severe malaria in other parts of the country is not known because of insufficient clinical research, inadequate laboratory resources and testing to confirm the diagnosis of malaria, and the absence of uniform clinical criteria of distinction between uncomplicated and severe malaria. Nonetheless, it has been estimated that uncomplicated malaria and severe malaria occur in 326.2 per 100,000 persons per year and 5.4 per 100,000 persons per year in Mauritania, respectively [21]. The prediction-biased proportion of severe cases treated as in-patients and incidence rate of malaria mortality were both estimated to be about 24–26 per 100,000 per year in Mauritania [21,22]. Severe malaria usually occurs in subjects with inadequate level of acquired immunity against malaria and those with delayed diagnosis and/or treatment. In endemic areas of intense and perennial transmission, as in many parts of sub-Saharan Africa, the population at risk of severe malaria includes young children and pregnant women [6]. Most adults in areas of intense and perennial transmission are generally thought to be protected against severe forms of malaria due to acquired immunity [23]. However, despite the fact that data on severe malaria in non-pregnant African adults living in endemic areas are limited, recent literature published in the 2010s suggests that severe and complicated malaria occurs in this population more frequently than previously thought in sub-Saharan Africa [19,20,24,25,26].

In southern Mauritania, malaria transmission is seasonal. Although the number of severe malaria cases was much lower in 2017, compared to 2016 and 2018, a similar pattern of severe malaria was observed in 2016–2019, with peak occurrence in October. This finding should alert the health authorities to prepare and concentrate medical interventions during this period of the year. In this region, approximately equal proportions of children and adults are seen with symptomatic, uncomplicated malaria in public health centres [1,27]. However, the proportion of adults and children with severe malaria cannot be determined at present due to the lack of reliable information on laboratory-confirmed malaria in the region. Most of the patients (67%) included in the present study were residing in suburban areas, where access to health care service is less favourable, delaying diagnosis and appropriate treatment. Self-medication prior to hospitalization was widely practiced by adult patients. This practice has been known to be widespread in Africa [28,29,30,31]. While there is some evidence that home management of malaria implemented by trained community health worker may limit or delay the onset of severe malaria [32,33], an unsupervised, haphazard practice of self-medication, including self-administration of inappropriate drugs, leads to erroneous dosage and treatment duration, doing more harm than good by delaying appropriate malaria management. In the present study, a large majority of patients consulted medical clinics in the private sector and, on the basis of presumptive diagnosis, treated with inappropriate antimalarial drugs (chloroquine, sulfadoxine-pyrimethamine) or low-dose quinine before hospitalization for severe malaria. Clinical manifestations of severe malaria in adults usually occur within 3–7 days after the initial onset of symptoms [6]. The present study showed that up to nine days passed since the onset of symptoms by the time the patients with severe malaria were admitted to the hospital. This diagnostic and therapeutic delay contributes to disease evolution towards severe malaria [26,30]. Easier access to health centres and rapid diagnostic and therapeutic interventions are clearly needed to prevent progression of severity.

The WHO recommends parenteral artesunate for at least 24 h followed by oral ACT to treat severe malaria [34]. The Mauritanian Ministry of Health recommends the administration of parenteral quinine for severe malaria, followed by ACT [11]. In Kiffa Regional Hospital, the national guideline was applied to treat most of the adult patients presenting with severe malaria. An intramuscular artemether was administered to patients presenting with haemoglobinuria, because quinine is contraindicated in patients with blackwater fever [6]. Despite the high efficacy of antimalarials used, the prognosis of severe malaria in adults was poor. In addition to delay in access to hospitals and treatment, the high mortality rate among patients with severe malaria is also probably related to the continued recommendation of the Mauritanian malaria control unit to administer parenteral quinine, without any loading dose, despite the WHO recommendation to treat severe malaria with parenteral artesunate (first choice) or intramuscular artemether (second choice) until oral ACT can be taken [6]. Intramuscular or intravenous infusion of quinine (loading dose followed by maintenance doses) is the third choice, in the absence of parenteral artesunate and artemether. Although the difference in treatment efficacy between quinine and artemether did not reach statistical significance, probably due to the small sample size, our data suggested a decrease in mortality rate among Mauritanian adults treated with artemether. Other studies have shown that artemisinin derivatives are superior to quinine in saving the lives of adults with severe malaria [6,35,36].

## 5. Conclusions

The results of the present study showed that mortality due to severe malaria in Mauritanian adults is high. Further reinforcement of preventive measures, prompt and reliable diagnosis, appropriate therapeutic intervention, and easier access to health centres, are required to lower mortality associated with malaria. Moreover, it is recommended that Mauritania follow the footsteps of other countries and adopt the WHO-recommended strategy to use parenteral artesunate as the first-line treatment for severe malaria.

## Figures and Tables

**Table 1:** Clinical Manifestations of Severe Malaria in Mauritanian Adult Patients.

Clinical Features	Number of Patients (%)			*p*-Value
	On Admission	Cured (n = 85)	Deceased (n = 14)	
Impairment of consciousness	81 (81.8)	67 (78.8)	14 (100)	NS *
Multiple convulsions (>2 episodes/24 h)	70 (70.7)	56 (65.9)	14 (100)	0.009
Cardiovascular collapse	61 (61.6)	49 (57.6)	12 (85.7)	NS *
Respiratory distress (acidotic breathing)	43 (43.4)	33 (38.8)	10 (71.4)	0.04
Severe anaemia (haemoglobin ≤ 80 g/L)	36 (36.4)	27 (31.8)	9 (64.3)	0.03
Haemoglobinuria	27 (27.3)	18 (21.2)	9 (64.3)	0.002
Acute renal failure (Cr > 265 µmol/L)	25 (25.3)	17 (20.0)	8 (57.1)	0.006
Hypoglycaemia (blood sugar < 0.4 g/L)	13 (13.1)	11 (12.9)	2 (14.3)	NS
Jaundice	12 (12.1)	6 (7.1)	6 (42.9)	0.002
Abnormal bleeding	7 (7.1)	3 (3.5)	4 (28.6)	0.007

Data present the number of patients with different clinical features on admission in patients who were cured and those who deceased. An impairment of consciousness denotes Glasgow coma score < 11. Cardiovascular collapse is defined as systolic blood pressure < 80 mm Hg. The *p*-values refer to the comparison of patients who were cured and those who died. Cr, creatinine; NS, non-significant (*p* > 0.05). * Statistically non-significant but *p* = 0.07.
